# Reproductive risk factors associated with breast cancer in women in Bangui: a case–control study

**DOI:** 10.1186/s12905-017-0368-0

**Published:** 2017-03-06

**Authors:** Augustin Balekouzou, Ping Yin, Christian Maucler Pamatika, Cavin Epie Bekolo, Sylvain Wilfrid Nambei, Marceline Djeintote, Komlan Kota, Christian Diamont Mossoro-Kpinde, Chang Shu, Minghui Yin, Zhen Fu, Tingting Qing, Mingming Yan, Jianyuan Zhang, Shaojun Chen, Hongyu Li, Zhongyu Xu, Boniface Koffi

**Affiliations:** 1 Department of Epidemiology and Biostatistics, School of Public Health, Tongji Medical College of Huazhong University of Sciences and Technology, Hangkong Road 13, Wuhan City, Hubei Province China; 2 Hospital Laboratory Friendship, Bangui, Central African Republic; 3 Ministry of Public Health, Centre Medical d’Arrondissement de Bare, Nkongsamba, Cameroon; 4 National Laboratory of Clinical Biology and Public Health, Bangui, Central African Republic; 5 Faculty of Health Sciences, University of Bangui, Avenue of the Martyrs, Bangui, Central African Republic

**Keywords:** Breast cancer, Reproductive factors, Women, Bangui, Central African Republic

## Abstract

**Background:**

Breast cancer (breast Ca) is recognised as a major public health problem in the world. Data on reproductive factors associated with breast Ca in the Central African Republic (CAR) is very limited. This study aimed to identify reproductive variables as risk factors for breast Ca in CAR women.

**Methods:**

A case–control study was conducted among 174 cases of breast Ca confirmed at the Pathology Unit of the National Laboratory in Bangui between 2003 and 2015 and 348 age-matched controls. Data collection tools included a questionnaire, interviews and a review of medical records of patients. Data were analysed using SPSS software version 20. Odd ratios and 95% confidence intervals (CI) for the likelihood of developing breast Ca were obtained using unconditional logistic regression.

**Results:**

In total, 522 women with a mean age of 45.8 (SD = 13.4) years were enrolled. Women with breast Ca were more likely to have attained little or no education (AOR = 11.23, CI: 4.65–27.14 and AOR = 2.40, CI: 1.15–4.99), to be married (AOR = 2.09, CI: 1.18–3.71), to have had an abortion (AOR = 5.41, CI: 3.47–8.44), and to be nulliparous (AOR = 1.98, CI: 1.12–3.49). Decreased odds of breast Ca were associated with being employed (AOR = 0.32, CI: 0.19–0.56), living in urban areas (AOR = 0.16, CI: 0.07–0.37), late menarche (AOR = 0.18, CI: 0.07–0.44), regular menstrual cycles (AOR = 0.44, CI: 0.23–0.81), term pregnancy (AOR = 0.26, CI: 0.13–0.50) and hormonal contraceptive use (AOR = 0.62, CI: 0.41–0.93).

**Conclusion:**

Breast Ca risk factors in CAR did not appear to be significantly different from that observed in other populations. This study highlighted the risk factors of breast Ca in women living in Bangui to inform appropriate control measures.

## Background

Breast cancer (breast Ca) is the most common cancer and the leading cause of cancer deaths among women worldwide [1]. Globally, every 3 min one woman is diagnosed with breast Ca, with a total of one million cases per year [2]. In 2012, the number of new cases diagnosed in women was 1,7 million (25% of all cancers), with 883,000 cases reported in developed countries against 794,000 in developing countries [1, 3]. In developed countries, breast Ca is the second most common cancer after cervical cancer [4]. Most studies on the risk factors for breast Ca were conducted in Caucasian populations.

A risk factor is defined as anything that increases your probability of developing breast Ca. However, on one hand, many of these risk factors are beyond individual’s control, such as sex, age, race, chest X-ray exposure, family history of breast Ca, personal history of breast Ca, pregnancy and breastfeeding. On the other hand, weight, diet, physical activity, smoking, alcohol, exposure to estrogen, use of oral contraceptives, stress and anxiety are called modifiable factors [5].

These lifestyles (eating habits, physical inactivity, smoking, alcohol consumption, obesity, etc.) as well as reproductive characteristics of women can also increase their risk of developing breast Ca [6]. It has been well established in the literature that changing reproductive patterns including late childbearing, low parity and shorter period of breastfeeding increase the risk of breast Ca [7]. Previous studies have also shown that, prolonged endogenous estrogen exposure owing to early menarche, late age at first delivery and late menopause or exogenous exposure, mainly due to hormone replacement therapy or use of oral contraceptive pills have been associated with breast Ca [8]. The role of certain factors such as spontaneous or induced abortion in the development of breast Ca remains controversial [8, 9].

Nulliparity, late age at first live birth and lack of breastfeeding are risk factors for breast Ca in developed countries. Reproductive factors play an important role in the development of breast Ca among women who lack access to good family planning in rich and poor countries [10].

What causes breast Ca? Why a double and even triple increase is seen in recent decades?

Indeed, women are now more likely to develop breast Ca than they were a decade ago. Survival rates have also increased. Nearly two in three women with breast Ca now survive the disease beyond 20 years, compared with less than half in the 1990s. More than three-quarters of women diagnosed with breast Ca survive for at least 10 years or more. All these increases were observed as a result of advances made in research. In Africa, this increasing incidence probably reflects the fact that nowadays, women live longer and adopt a lifestyle that favours high incidence rates (for example; decreased fertility, obesity, etc…). A large proportion of breast Ca in Africa has been observed in pre-menopausal women compared with those in Western countries, possibly reflecting the role of some specific risk factors [11]. The burden of breast Ca in Africa has been aggravated by lack of and limited access to standardised programs for cancer awareness, diagnosis and treatment [11, 12].

In recent decades, while the Central African Republic (CAR) began recording a significant reduction of infectious diseases through various national programs implemented, new diseases, including cancer and other non-communicable chronic diseases began emerging as new public health priorities [13]. Unfortunately, only few hospital studies had been conducted in this domain, and none had studied the risk factors associated with this disease in the CAR population [14, 15].

As part of a larger effort to inform the Ministry of Health on possible interventions to prevent breast Ca, this case–control study was conducted to determine the relationship between breast Ca and reproductive factors in women living in Bangui, CAR. The results of this study will help the Ministry of Health to develop new strategies for prevention, early diagnosis and treatment.

## Methods

We conducted a case–control study at the pathology unit of the National Laboratory, and at the general surgery and gynecology services of two tertiary care institutions in Bangui (CAR).

### Study population

Cases were identified among women with histologically confirmed breast Ca between September 2003 and September 2015. Controls were randomly recruited among women who came for other conditions unrelated to cancer at the National Laboratory of Bangui. For each case, two controls were selected. All controls were free of any cancer. They were matched for age, because breast Ca is an age-related disease and increasing age is the single most important risk factor after female gender [16]. In addition, all controls were considered to come from the same catchment area as the cases. The women came from various ethnic and socioeconomic backgrounds and thus represented the diversity of the CAR’s population.

### Inclusion criteria

All consenting women aged ≥ 15 years, living in Bangui, and who presented with histologically confirmed breast Ca between 2003 and 2015.

### Data collection

Data was collected from a cancer register of the pathology unit of the National Laboratory and from medical records of patients seen at the general surgery and gynecology services in Bangui. The risks and benefits of the study were explained to all eligible participants. Those who agreed signed an informed consent form before the interview. This interview was conducted in Sango (second official language in CAR). For participants who did not understand Sango, adult relatives interpreted the content of the questionnaire and consent form for better understanding. For minors or children, a written consent was obtained from close relatives or caretakers before being enrolled in the study. Each potential participant had the choice to accept or refuse to participate in the study. Questions were also granted from volunteers who wish clarification. For cases who had died, their relatives were selected as next of kin to provide data relating to their lifestyle.

### Study variables

The following explanatory variables were considered as reproductive factors: age, occupation, economic status, education level, areas of residence (urban or rural), ethnic group and marital status. In addition, age at first menarche, menstrual cycle frequency, dysmenorrhoea, full-term pregnancy, age at first live birth, abortion, parity, breastfeeding, menopausal status and use of hormones (hormonal replacement therapy or contraceptive pills).

Age was recoded as age groups. Their occupation was classified as a homemaker or paid employment outside of the home. Economic status was defined in terms of family income according to international poverty threshold. Low income if below 2 dollars a day, moderate between 2 and 4 dollars, good between 5 and 10 dollars and excellent above 10 dollars [17]. Place of residence was urban for those living in Bangui and rural for those living in other provinces. Their level of education was classified as illiterate, elementary, high school and university. Marital status was classified as married and single (including: divorced and widow). Menarche was defined as the age at which the first menses occurred. Menstrual cycle frequency was defined as regular or irregular. Dysmenorrhoea was defined as menstrual pain. Age at the first live birth was defined as the age when the first full-term birth (≥37 weeks of gestation) occurred. Abortion has been defined as termination of pregnancy before 28 weeks of gestation. Parity was determined by the number of pregnancies that a participant had before the diagnosis (of cases) or interview (of controls). For breastfeeding it was assessed whether or not it was practised and for how long. The menopausal status was defined as a complete cessation of menstruation in women before diagnosis (cases) or interview (control). Use of hormonal agents and their duration were assessed in women before diagnosis or interview.

### Statistical analysis

Pearson chi-square (*χ*2) test or Fisher’s exact test were used to compare the frequency distribution of categorical variables while the student *t*-test were used to compare the mean values for continuous variables between cases and controls. Unconditional logistic regression models were used to estimate odd ratios (OR) and their 95% confidence intervals (CI) for the association between reproductive factors and breast Ca. Variables associated with breast Ca at significance level below 0.2 in the univariate analysis were included in the multivariate model. Variables associated with breast Ca at the significant level below 0.05 were kept in the multivariate model following backward elimination. Results were presented as adjusted odds ratio (AOR), 95% CI and *P* values. All analyses were performed using Statistical Package for Social Sciences (SPSS Inc., Chicago, IL, USA) version 20.

## Results

In total, 174 cases and 348 age-matched controls were included. The response rate was 85.99% (522/607). The age at diagnosis for the cases ranged from 16 to 90 years with a mean of 45.83 (SD = 13.5) years. The mean age for the control was 45.79 (SD = 13.3) years.

### Socio-demographic characteristics

Table 1 shows the socio-demographic characteristics for cases and controls. There were significant differences between cases and controls with respect to occupation (*p* = 0.001), economic status (*p* = 0.01), education level (*p* < 0.001), area of residence (*p* <0.001), marital status (*p* <0.001) and parity (*p* = 0.008). Over 69% (121/174) of the cases as compared to 82% (287/348) of controls were housewives with a moderate economic status (56.9 and 66.4%). Nearly 13% (23/174) and 14% (51/348) of the cases and controls, respectively, had attained higher level of academic study and lived in cities (85.6 and 96.9%). Unmarried women made up 75.9% (132/174) of cases against 89.9% (313/348) of controls. A small proportion of cases (17.9%) and controls (9.8%) were nulliparous.

**Table 1:** Socio-demographic characteristic of study participants

Variables	Cases (174) Freq (%)	Controls (348) Freq (%)	Total (522) Freq (%)	*χ*2	*p*-value
Age group					
15–39	57 (32.8)	112 (32.2)	169 (32.4)		
40–44	25 (14.4)	54 (15.5)	79 (15.1)		
45–49	18 (10.3)	34 (9.8)	52 (10.0)		
50–54	33 (19.0)	68 (10.5)	101 (19.3)		
55–59	15 (8.6)	30 (8.6)	45 (8.6)		
60–64	10 (5.7)	18 (5.2)	28(5.3)		
65–69	5 (2.9)	12 (3.4)	17 (3.3)		
70–74	6 (3.4)	10 (2.9)	16 (3.1)		
≥ 75	5 (2.9)	10 (2.9)	15 (2.9)		
Mean age (SD)	45.83 (13.55)	45.79 (13.34)	45.80 (13.40)	t = -0.035	0.97^a^
Occupation				11.36	*0.001* ^**b**^
Housewife	121 (69.5)	287 (82.5)	408 (78.2)		
Employed	53 (30.5)	61 (17.5)	114 (21.8)		
Economic status				9.92	*0.01* ^**c**^
Poor	24 (13.8)	56 (16.1)	80 (15.3)		
Moderate	99 (56.9)	231 (66.4)	330 (63.3)		
Good	48 (27.6)	55 (15.8)	103 (19.7)		
Excellent	3 (1.7)	6 (1.7)	9 (1.7)		
Education level				22.2	*0.000* ^**c**^
Illiterate	45 (25.9)	34 (9.8) 34(9.7)	79 (15.1)		
Elementary	57 (32.7)	144 (41.4)	201 (38.5)		
High School	49 (28.2)	119 (34.2)	168 (32.2)		
University	23 (13.2)	51 (14.7)	74 (14.2)		
Residence				21	*0.000* ^**b**^
Urban	149 (85.6)	336 (96.6)	485 (92.9)		
Rural	25 (14.4)	12 (3.4)	37 (7.1)		
Marital status				18.28	**0.000** ^**c**^
Married	42 (24.1)	32 (10.1)	77 (14.8)		
Single	132 (75.9)	313 (89.9)	445 (85.2)		
Parity				9.6	**0.008** ^**c**^
Nulliparus	31 (17.9)	34 (9.8)	65 (12.5)		
1–2	46 (26.6)	128 (36.8)	174 (33.4)		
≥ 3	96 (55.5)	186 (53.4)	282 (54.1)		

Frequency was calculated by using Cross tabulation analyze. Employee includes all sectors: public and private. Poor economic status (income < 2 dollars a day), moderate (income = 3 to 4 dollars a day), good (income = 5 to 10 dollars per day) and excellent (income > 15 dollars a day); Residence: Town (Bangui) and Rural (outside Bangui)

Legend: Frequency; *χ2* chi square, *SD* standard deviation

*χ*2 was calculated by using Fisher’s exact chi square test

^a^ *p*-value was calculated by using *T*-test

^b^ *p*-value was calculated by using Pearson’s chi square test

^c^ *p*-value was calculated by using Fisher’s exact chi square test, *p*-value < 0.05 in italic

### Socio-demographic factors and their association with breast cancer

The odd ratios for the association between socio-demographic factors and breast Ca were summarised in Table 2. The odds of breast Ca were 11.23 and 2.40 times higher (95% CI: 4.65–27.14, *p* <0.001 and 95% CI: 1.15–4.99, *p* = 0.01) among women with little or no education compared with those with university education. The odds of breast Ca were 2.09 times higher among married women compared with singles (95% CI: 1.18–3.71, *p* =0.01). Women in employment and those living in cities showed decreased odds of 0.32 (95% CI: 0.19–0.56, *p* < 0.001) and 0.16 (95% CI: 0.07–0.37, *p* < 0.001), compared with housewives and those living in rural areas, respectively.

**Table 2:** Socio-demographic factors and their association with breast cancer

Factors associated	Cases (174) N (%)	Controls (348) N (%)	Univariate analysis			Multivariate analysis		
			COR	95%, CI [L-U]	*p*-value	AOR	95%, CI [L-U]	*p*-value
Occupation								
Housewife	121 (69.5)	287 (82.5)	1.00 (Ref)			1.00 (Ref)		
Employer	53 (30.5)	61 (17.5)	*0.31*	[0.31–0.74]	*0.001*	*0.32*	[0.19–0.56]	*0.000*
Education level								
Illiterate	45 (25.9)	34 (9.8)34 (9.7)	*2.93*	[1.51–5.70]	*0.001*	*11.23*	[4.65–27.14]	*0.000*
Elementary	57 (32.7)	144 (41.4)	*1.87*	[1.49–4.56]	*0.02*	*2.40*	[1.15–4.99]	*0.01*
High School	49 (28.2)	119 (34.2)	0.91	[0.50–1.65]	0.76	1.76	[0.86–3.61]	0.12
University	23 (13.2)	51 (14.7)	1.00 (Ref)			1.00 (Ref)		
Residence								
Urban	149 (85.6)	336 (96.6)	*0.21*	[0.10–0.43]	*0.000*	*0.16*	[0.07–0.37]	*0.000*
Rural	25 (14.4)	12 (3.4)	1.00 (Ref)			1.00 (Ref)		
Marital status								
Married	42 (24.1)	32 (10.1)	*2.84*	[1.73–4.65]	*0.000*	*2.09*	[1.18–3.71]	*0.012*
Single	132 (75.9)	313 (89.9)	1.00 (Ref)			1.00 (Ref)		

Legend: *L* lower, *U* upper, *COR* crude odds ratio, *AOR* adjusted odds ratio, *Ref* reference, *CI* confidence interval

OR was calculated by using logistic regression​, *p*-value< 0.05 in italic

**Table 3:** Reproductive factors and their association with breast cancer

Factors associated	Cases (174) N (%)	Controls (348) N (%)	Univariate analysis			Multivariate analysis		
			COR	95%, CI [L-U]	*p*-value	AOR	95%, CI [L-U]	*p*-value
Age at menarche (year)								
< 12	8 (4.6)	62 (17.8)	1.00 (Ref)			1.00 (Ref)		
≥ 12	166 (95.4)	286 (82.2)	*0.22*	[0.10–0.47]	*0.000*	*0.18*	[0.07–0.44]	*0.000*
Menstrual cycles								
Regular	142 (81.6)	312 (89.7)	*0.51*	[0.30–0.85]	*0.01*	*0.44*	[0.23–0.81]	*0.009*
Irregular	32 (18.4)	36 (10.3)	1.00 (Ref)			1.00 (Ref)		
Term pregnancy								
Yes	143 (82.2)	319 (91.7)	*0.41*	[0.24–0.72]	*0.002*	*0.26*	[0.13–0.50]	*0.000*
No	31 (17.8)	29 (8.3)	1.00 (Ref)			1.00 (Ref)		
Abortion								
Yes	114 (65.5)	99 (28.4)	*4.77*	[3.23–7.05]	*0.000*	*5.41*	[3.47–8.44]	*0.000*
No	60 (34.5)	249 (71.6)	1.00 (Ref)			1.00 (Ref)		
Parity								
Nulliparous	31 (17.9)	34 (9.8)	*1.76*	[1.02–3.04]	*0.04*	*1.98*	[1.12–3.49]	*0.01*
1–2	46 (26.6)	128 (36.8)	0.69	[0.45–1.05]	0.69	0.76	[0.49–1.17]	0.2
≥ 3	96 (55.5)	186 (53.4)	1.00 (Ref)			1.00 (Ref)		
Mode of Breastfeeding^a^								
Natural	131 (91.6)	289 (91.2)	*0.20*	[0.04–0.85]	*0.03*	0.33	[0.02–1.15]	0.43
Artificial	4 (2.8)	20 (6.3)	0.45	[0.16–1.23]	0.12	0.51	[0.10–2.55]	0.41
Mixed	8 (5.6)	8 (2.5)	1.00 (Ref)			1.00 (Ref)		
Use of hormonal contraceptive								
Yes	50 (28.9)	143 (41.1)	*0.58*	[0.39–0.86]	*0.01*	*0.62*	[0.41–0.93]	*0.02*
No	123 (71.1)	205 (58.9)	1.00 (Ref)			1.00 (Ref)		
Duration of using hormone contraceptive (year)^b^								
≤ 5	36 (72.0)	103 (72.1)	1.00 (Ref)			NA		
> 5	14 (28.0)	40 (27.9)	0.97	[0.47–1.99]				

Legend: *L* lower, *U* upper, *COR* crude odds ratio, *AOR* adjusted odds ratio, *Ref* reference, *CI* confidence interval, *NA* not applicable

OR was calculated by using logistic regression, *p*-value < 0.05 in italic

^a^breastfeeding and duration of breastfeeding were calculated bases the number of births, ^b^duration of using hormonal contraceptive was calculated bases the number of the women who use hormonal contraceptive

### Reproductive factors and their association with breast cancer

The odds of breast Ca were 5.41 times higher among women with a history of abortion compared with those with none (95% CI: 3.47–8.44, *p* <0.001). Nulliparous women showed a 1.98 times the odds of breast Ca (95% CI: 1.12–3.49, *p* = 0.01), compared with women with one or more children. Women with late menarche (≥12 years old) and those who had regular menstrual cycles were found to have decreased odds of 0.18 (95% CI: 0.07–0.44, *p* < 0.001) and 0.44 (95% CI: 0.23–0.81, *p* = 0.009) respectively, compared with those with early menarche (< 12 years old) and irregular menstrual cycles (Table 3). Similarly, for women who had term pregnancies, used hormonal contraceptives and practiced natural breastfeeding were significantly associated with lower odds of having breast Ca by 0.26 (95% CI: 0.13–0.50, *p* <0.001), 0.62 (95% CI: 0.41–0.93, *p* = 0.02) and 0.20 (95% CI: 0.04–0.85, *p* = 0.03) respectively, compared to those who did not (Table 3). The association between breastfeeding and breast Ca was not statistically significant (AOR =0.03, 95% CI: 0.02–1.15, *p* = 0.43).

## Discussion

The risk factors for breast Ca examined in this study are part of the many known key drivers in populations with low incidence including Africa. However, certain risk factors are not compatible with breast Ca according to consensus indications [18]. There is an international variation in incidence of breast Ca whose reason remains unclear. Given the emerging picture of the biological and epidemiological disparities in breast Ca between countries with high and low income, there will often be a need to re-use these associations between breast Ca and risk factors already known and / or suspected newly [19].

In this study, we have uncovered commonly known risk factors associated with breast Ca among women in CAR. With regards to educational level and its association with breast Ca, our study found that breast Ca was more common among less educated than in more educated ladies. There is an agreement with findings from a population-based cohort, between 1964–2008 in Israel in 2015 [20], but in contrast to the gradient effect observed in European populations during the 1990s [21]. One explanation for this might be the small number of women with a university education in our study.

According to previous studies, socio-economic status has been shown to be a strong predictor of health status [22]. Indeed, socio-economic inequalities could affect the time of diagnosis, survival or mortality due to cancer despite improved knowledge, reduction of risk factors for cancer, early diagnosis and treatment [23]. The results of this study indicated that employment has a significant protective effect on breast Ca. This observation is inconsistence with the study in Iran in 2015, which focused on the socio-economic levels of the family as effective critical risk factors for breast Ca among Iranian women [24]. Our results could have the explanation that employed women generally have more family income to afford health insurance. In addition, the economic environment also could affect the willingness of a person to spend money on her medical needs.

Our study found that, living in an urban environment decreased the risk of developing breast Ca. We expected that there would be differences between rural and urban areas because of perceived differences in lifestyle in terms of diet and environmental factors. Our results are in agreement with the study conducted in India in 2014 which showed that people living in urban area were better protected compared those in the rural area [25]. On the other side, a recent study in Uganda in 2016, has suggested no significant urban-urban difference in breast Ca risk [26]. A plausible explanation in CAR could be linked to the fact that the lone diagnosis laboratory for the diagnosis of breast Ca being located in Bangui, meaning access for people living in the provinces was very limited. This could justify the low prevalence of cases reported among women living in rural areas compared to those living in Bangui. Furthermore, in view of advanced cancer stage of our patients during the diagnosis and the status of women coming for other conditions which were considered as controls in the study, it is certain that the results concerning variables such as employment, education level and live in an urban area could be influenced by selection bias.

Our study showed that the risk of breast Ca increases among married women compared with the unmarried. Our results corroborate with studies conducted in Iran (2002) and India (2013), which reported a higher risk for married women [27, 28], but some authors have reported no significant correlation between risk of breast Ca and marriage [29–32] . Other studies on health, disease and mortality have shown that marriage is a protective factor for breast Ca outcomes [33, 34]. It is important to cautious about the quality of the marital relationship reported prior to or following the diagnosis of breast Ca.

During the last decades in the CAR, marriage has been closely linked to the socio-economic status of the future couple and /or their respective families. Unfortunately, only a minority group (20%) of women in this study belongs to the high socio-economic class, with improved access to medical care. This situation makes us believe that marriage is an indication of good socio-economic status. It is important to note that, following the results of this study, demonstrating marriage as a risk factor for breast Ca, we think that these results could have been influenced by selection bias. Moreover, in a context were the woman lacks the partner’s moral support, marriage can harm her well-being and health outcomes; support from others (family or friends) can have a stronger influence on the survival of the patient than the support of a spouse.

The results of our study showed that the odds of breast Ca were lower among women who had their menarche after the age of 12 years. Our results contrast with previous studies conducted in Northeast Brazil that have revealed a positive association between early menarche and breast Ca risk [35]. However, a study in Malaysia has shown that age at menarche was not a risk factor for breast Ca [36].

According to the literature, an ovulatory menstrual cycles may have a protective effect on breast Ca [37]. However, an epidemiological study conducted in Nigeria showed conflicting trends regarding the association between dysfunctional ovulatory cycles and breast Ca risk [38]. Differences may be due to genetic factors or lifestyle in the populations studied, or to methodological limitations of some studies. The definition of ovulatory cycle varied across studies and included extended of cycles, continuous irregularity, amenorrhea and infertility. Our study showed that regular menstrual cycles had a protective effect against breast Ca. This results corroborate with studies by Lecarpentier et al.(2015) that focused on the long duration of ovulatory cycles (> 31 days) as an increased risk for breast Ca in women [39].

Some studies consider a high number of pregnancies as a protective factor against breast Ca. A study conducted in Denmark reported that more pregnancies reduce the risk of the disease [40]. Our results are consistent with the study conducted in North-West of Iran, that demonstrating the protective effect of pregnancy among women [4]. Various studies worldwide, have considered the factor “abortion” as another important factor for breast Ca [24, 41]. From our analysis, the odds of breast Ca were higher among women who have had an abortion compared to those who had not. These results corroborate with the study conducted by Lipworth et al., in 1995, which showed that induced abortion before or after first full-term pregnancy increased the odds of breast Ca among women in Greece by 2.06 and 1.59, respectively [42]. However, it is important to note that our study presented significant conceptual limitations that could have affected the results. Some of the main limitations were the small number of women included in the study, data were collected only after breast Ca was diagnosed and the history of abortion was based on self-reporting rather than on their medical records, which could have introduce selection bias. On the other hand, prospective studies, which are more rigorous in design and not affected by such bias, have consistently shown no association between induced abortion and breast Ca risk [43–46].

According to the literature, parity is one of the best established and modifiable factors involved in breast Ca among women [47]. Women above 25 years old have an increased risk immediately after parturition due to inflammatory processes that occur in breast tissue during her development of postpartum [48]. Despite this initial increase, the overall risk of life parous women remains significantly reduced [47]. A recent study shows a significant contribution of nulliparity in the increased risk of breast Ca. Our study showed that breast Ca was more likely to be reported among nulliparous women than women who have more than three children. This finding is consistent with recent studies worldwide [36, 49].

Several studies have highlighted the effect of modifiable lifestyle factors such as breastfeeding on the risk of breast Ca. The majority of these reported a protective effect of breastfeeding on breast Ca in pre-menopausal women [25, 50–52]. The protective effect of breastfeeding is thought to occur through differentiation of breast tissue and reduction in the number of lifetime ovulatory cycles [53]. Our results showed that the protective effect of breastfeeding is an independent predictor of breast Ca. Indeed, the majority of our study population has multiple live births, they almost always breastfed their babies, for more than 12 months or longer per child. This was consistent to recent studies carried out in different populations which also found a protective effect of breastfeeding [25, 50–54].

With regards to the use of hormonal contraceptive pills and their association with breast Ca, our study showed some protective effect with OR of 0.62. This is comparable with some previous studies conducted in Iran (2008) and Pakistan (2015) which also showed OR of 0.41 and 0.92 respectively [55, 56]. However other few studies conducted in China (1992), Norway and Sweden (2002) and Malaysia (2005), showed positive association of breast Ca with use of hormonal contraceptive pills [57–59]. According to the principle of preventive medicine, use of contraceptive hormone is recommended to enforce family planning policy (child spacing), because in Africa and in some other developing countries there is no law limiting the number of children per family. However, when referring to oral contraceptive pills use, according to some authors its use is only recommended for women who are procreative (able to have children), because their use appears to slightly increase the risk of cancer for a limited period. Women who stopped using oral contraceptives over a 10-year period do not appear to have an increased risk of breast Ca [60]. Unfortunately our results did not show a significant risk between the duration of contraceptive use and breast Ca.

Some limitations must be considered to explain the findings of this study. Firstly, the study was carried out on a small of population in CAR; therefore known risk factors may be different in the general population of Central Africa. Secondly, information (recall) bias from self-reporting and from relatives. Using women who came for other conditions as controls introduced some selection bias in the study, given that cancer shares some risk factors with other non-communicable diseases. However, the results and limitations of the study are very useful due to the fact they contribute to the ongoing research in the field of breast Ca in CAR. In addition, this study was conducted in a developing country where changes in lifestyle can provide other important information about breast Ca risk factors.

## Conclusion

Breast Ca risk factors in CAR do not appear to be significantly different from those observed in other populations. This study highlighted the risk factors of breast Ca women living in Bangui to inform appropriate control measures. Other more extensive studies are needed to investigate other unknown determinants of breast Ca.

## Abbreviations

AOR: Adjusted odds ratio
Breast Ca: Breast cancer
CAR: Central African Republic
CI: Confidence interval
COR: Crude odds ratio
HIV: Human Immunodeficiency Virus
SD: Standard deviation
SPSS: Statistical Package for Social Sciences
SSA: Sub-Saharan African
WHO: World Health Organization
